# A multi-scale detection model for tomato leaf diseases with small target detection head

**DOI:** 10.3389/fpls.2025.1598534

**Published:** 2025-09-16

**Authors:** Hao Sun, Xiaofeng Li, Xiaofang Li, Xuewei Wang, Zhenqi Cheng, Mohammed Abdulhakim Al-Absi, Liqun Xiao, Rui Fu

**Affiliations:** ^1^ Shandong Facility Horticulture Bioengineering Research Center, Weifang University of Science and Technology, Weifang, China; ^2^ Department of Smart Computing, Kyungdong University, Goseong-gun, Gangwon-do, Republic of Korea; ^3^ School of Computer, Sichuan Technology and Business University, Chengdu, China

**Keywords:** tomato disease detection, deep learning, multi-scale detection, serial multi-kernel feature aggregation, symmetrical re-calibration aggregation, FPN

## Abstract

In tomato cultivation, various diseases significantly impact tomato quality and yield. The substantial scale differences among diseased leaf targets pose precise detection and identification challenges. Moreover, early detection of disease infection in small leaves during the initial growth stages is crucial for implementing timely intervention and prevention strategies. To address these challenges, we propose a novel tomato disease detection method called TomatoLeafDet, which integrates multi-scale feature processing techniques and small object detection technologies.Initially, we designed a Cross Stage Partial -Serial Multi-kernel Feature Aggregation (CSP-SMKFA) module to extract feature information from targets at different scales, enhancing the model's perception of multi-scale objects. Next, we introduced a Symmetrical Re-calibration Aggregation (SRCA) module, incorporating a bidirectional fusion mechanism between highresolution and low-resolution features. This approach facilitates more comprehensive information transmission between features, further improving the efficacy of multi-scale feature fusion. Finally, we proposed a Re-Calibration Feature Pyramid Network with a small object detection head to consolidate the multi-scale features extracted by the backbone network. This network provides richer multi-scale feature information input for detection heads at various scales. Results indicate that our method outperforms YOLOv9 and YOLOv10 on two datasets. Notably, on the CCMT tomato dataset, the proposed model achieved improvements in mean Average Precision (mAP50) of 4.4%, 1.9%, and 2.3% compared to the baseline model, YOLOv9s, and YOLOv10n, respectively, exhibiting significant efficacy.

## 1 Introduction

Tomatoes are one of the most widely consumed vegetables globally, and they are esteemed for their versatility and high nutritional value. Rich in vitamins, minerals, and antioxidants, tomatoes play a crucial role in the balanced diet of most households. However, in recent years, tomato cultivation has faced significant challenges due to increasingly unstable environmental conditions resulting from climate change. These unpredictable weather patterns have led to a surge in various leaf diseases and pest infestations, particularly during the early stages of tomato plant growth Redmond et al. (2018). Common diseases such as leaf spot, leaf blight, and yellow leaf curl have become more prevalent, severely impacting both the yield and quality of tomato crops Liu and Wang (2021). The rising incidence of diseases has prompted farmers to increase their reliance on pesticides as a defensive measure. However, this approach has also brought about a series of problems. For instance, excessive use of pesticides may lead to the development of pesticide resistance in tomato plants, necessitating the application of different and more potent, expensive pesticides. This vicious cycle increases the economic burden on farmers and poses a serious threat to ecosystems. Furthermore, pesticide residues on tomato surfaces may significantly reduce the nutritional value of the fruit and potentially endanger consumer health Amr and Raie (2022).

**Figure 1:**
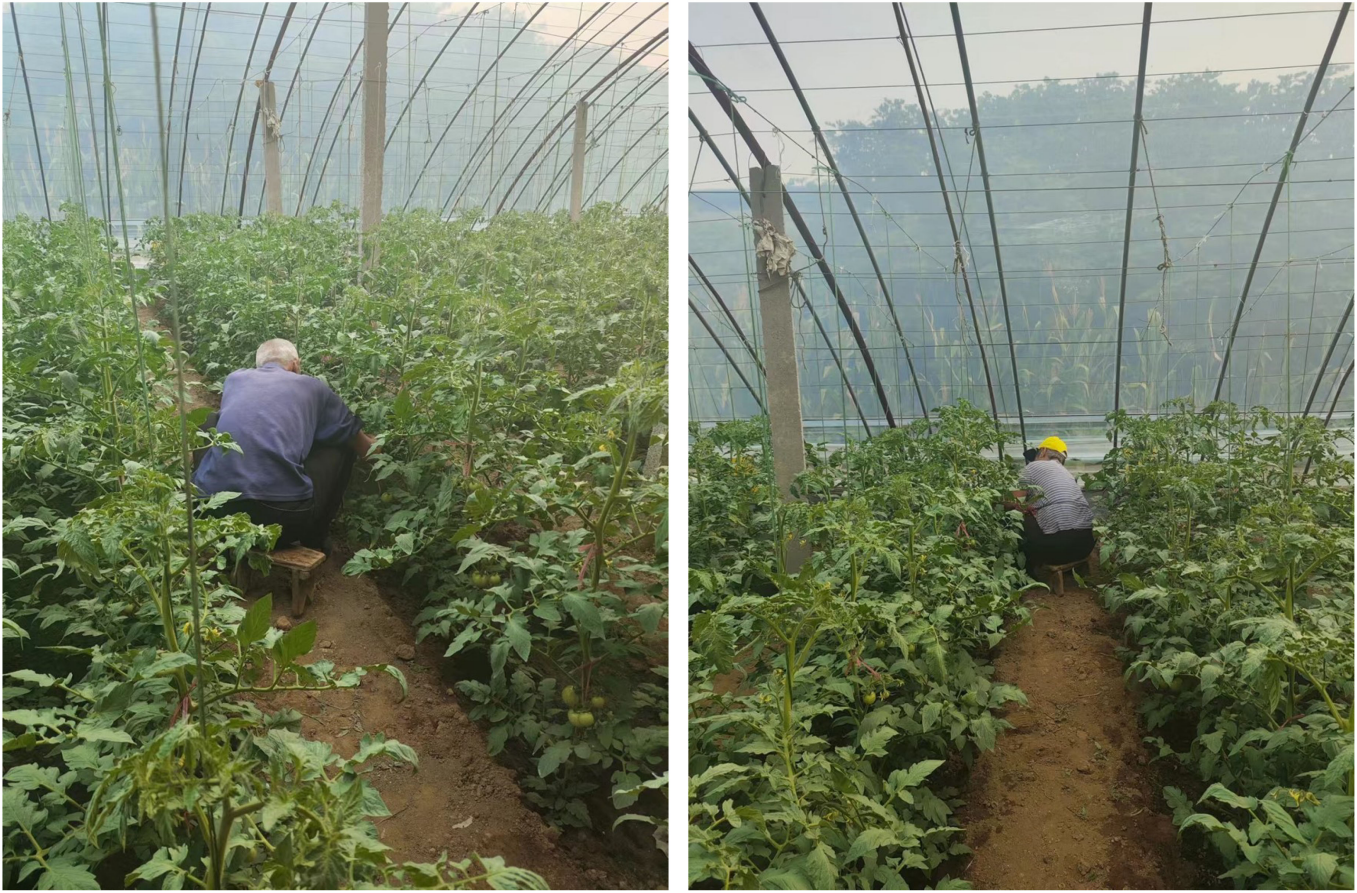
The current status of tomato cultivation.

Early intervention and prevention strategies are crucial for mitigating these issues. Farmers can significantly reduce pesticide use, minimize expenses, and ensure better crop yield and quality by detecting and addressing diseases initially. This approach protects the nutritional integrity of tomatoes and enhances the economic benefits of tomato cultivation. However, implementing effective early intervention strategies requires accurate and timely leaf disease detection methods, which have proven to be a formidable challenge in agricultural practices, as illustrated in **Figure 1**. Traditional manual inspection methods for identifying tomato leaf diseases have numerous limitations; these methods are labor-intensive, time-consuming, and often inefficient Shoaib et al. (2023). Furthermore, the subtle symptoms of early-stage diseases can be easily overlooked even by experienced professionals, leading to delayed interventions and increased vegetable losses Demilie (2024). The need for more advanced, reliable, and efficient detection methods has become increasingly evident in modern agriculture.

The emergence of deep learning techniques has opened a new phase for vegetable disease detection methods. Convolutional Neural Networks (CNNs) have provided promising solutions for vegetable disease classification. Various CNN-based models have been proposed and applied in agricultural environments, demonstrating significant advantages over traditional methods. However, these initial deep learning approaches primarily focused on classification tasks Ferdinand and Al Maki (2022); Madhav et al. (2021), failing to address the crucial issue of disease localization. This limitation hindered their ability to fully replace manual inspection processes, as precise location information is essential for targeted treatment and intervention strategies. The latest advances in computer vision have facilitated the development of faster and more accurate object detection models. These models can classify diseases and precisely locate the diseased areas in images, marking a significant leap in automated plant disease detection. Object detection models can be broadly categorized into two types: two-stage detectors and single-stage detectors. The Faster R-CNN Ren et al. (2015) model is particularly prominent among two-stage models. The methods divide the task of identifying objects into two key steps: the first step involves producing potential areas or bounding boxes that might contain objects of interest; the second step focuses on categorizing these candidate regions and determining their exact positions He et al. (2023); Zhao et al. (2022); Wang et al. (2022b). In contrast, single-stage detectors, especially the YOLO (You Only Look Once) Redmon et al. (2016); Redmon and Farhadi (2016); Redmon and Farhadi (2018); Bochkovskiy et al. (2020); Ultralytics (2022); Wang et al. (2022a); Wang et al. (2024); Li et al. (2022); Jocher et al. (2023) series models, have garnered attention due to their efficiency and accuracy. Researchers have also begun to focus on improving the performance of YOLO models in vegetable disease detection. For instance: Wang et al. (2021) proposed a YOLOv3 detection model incorporating a novel IOU by enhancing tomato pest and disease sample data Wang et al. (2021). Liu et al. (2022) proposed a novel YOLOv4 detection model for tomato pests by integrating three sets of attention mechanisms Liu et al. (2022). Liu et al. (2023) developed a YOLOv5 model with a novel loss function to address the detection of tomato brown rot Liu et al. (2023). Wang et al. (2024) integrated Transformer into YOLOv8 to improve tomato disease detection, enhancing the model’s ability to capture disease detail features Wang and Liu (2024). Jiang et al. (2024) combined Swin Transformer and CNN to optimize YOLOv8’s feature extraction capability, improving the model’s detection ability for cabbage diseases in complex environments Jiang et al. (2024). Liu and Wang (2024) proposed a multi-source information fusion method based on YOLOv8 to improve the accuracy of multi-vegetable disease detection Liu and Wang (2024).

**Figure 2:**
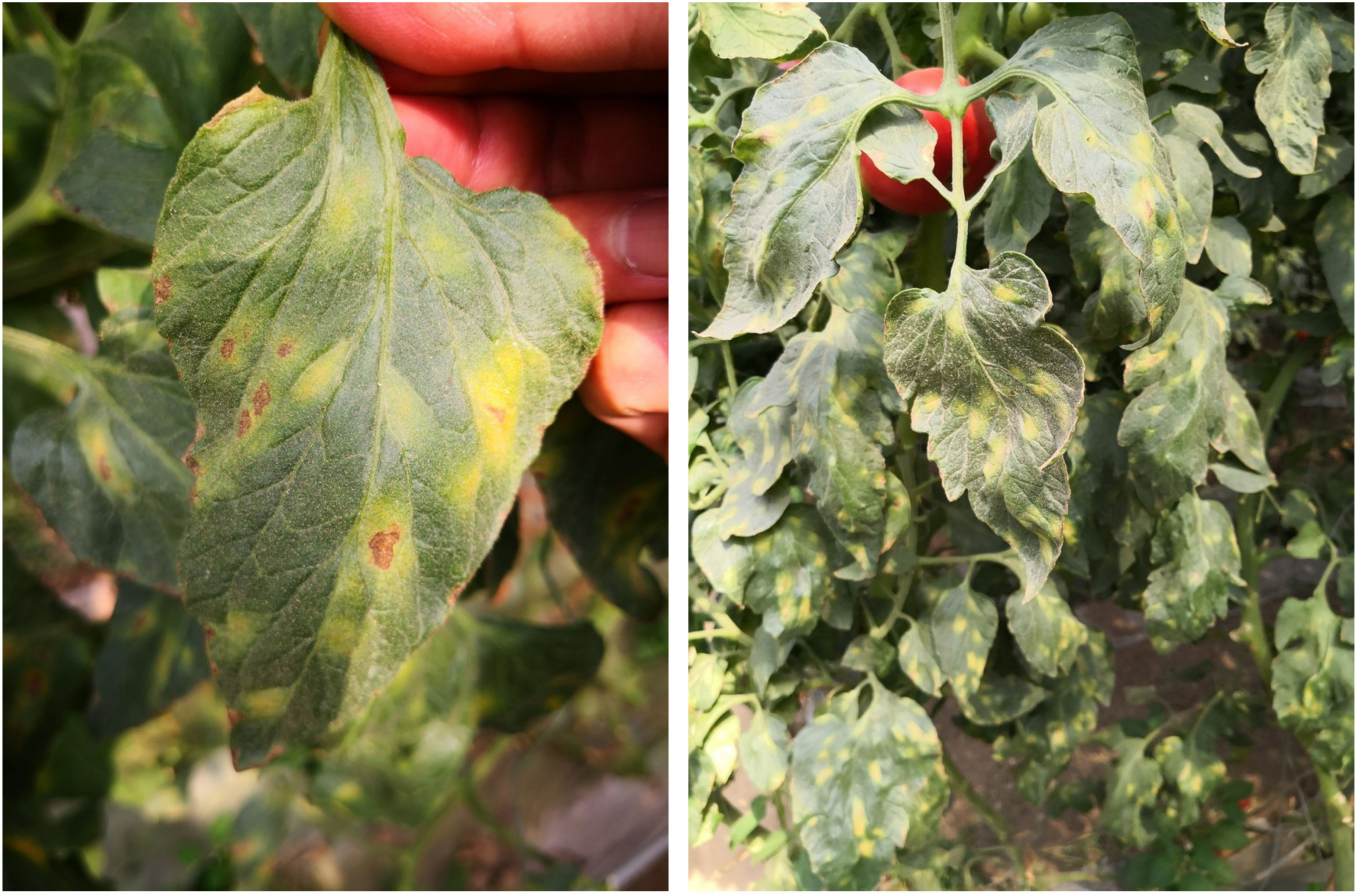
Sample Data Collected from Tomato Leaves at Different Scales.

Although these improved YOLO models address some issues in crop disease detection, they primarily focus on solving problems such as small sample sizes, complex background environments, and model parameter optimization. However, the impact of leaf size variation on detection accuracy has rarely been addressed or mentioned. As shown in **Figure 2**, some data samples are collected from one or two leaves, while others are collected from multiple leaves. Despite having the same sample size, the scale of the target data varies significantly. Most existing models have been optimized to detect diseases on leaves of normal size, potentially overlooking early infections on smaller leaves. This limitation is particularly important in the context of early intervention strategies. Therefore, it is crucial to be able to specifically perceive diseases on leaves of various sizes, especially smaller ones. To address this critical gap in current tomato disease leaf detection methods, we propose a novel tomato disease leaf detection algorithm, TomatoLeafDet. The contributions of this study can be summarized as follows:

- We propose a real-time detection model named “TomatoLeafDet”. By integrating various novel multiscale strategies, this model aims to simultaneously capture and process features of leaves of various sizes, with particular emphasis on improving the detection capability for smaller leaves.
- We propose a Cross Stage Partial - Serial Multi-kernel Feature Aggregation (CSP-SMKFA) module, which introduces a serial multi-kernel convolutional network capable of extracting multi-scale feature information from the input. It combines 1x1 convolutional layers and residual connections to fuse features of different scales, enhancing the model’s expressive ability.
- We propose a Symmetrical Re-calibration Aggregation (SRCA) module. Through an adaptive attention mechanism, it adaptively adjusts the weights of features according to the different resolutions and contents of feature maps, thereby better capturing the multi-scale features of the target.
- Utilizing the CSP-SMKFA and SRCA modules, we propose a Re-Calibration Feature Pyramid Network (Re-CalibrationFPN) with a small target detection head. It introduces a bidirectional fusion mechanism between high-resolution and low-resolution features, enabling more comprehensive information transmission between features and further enhancing the effect of multi-scale feature fusion. Simultaneously, it incorporates a specialized small target detection head aimed at improving the sensitivity and accuracy of disease detection on smaller leaves.
- Through comparative and ablation experiments, our model demonstrates significant advantages compared to the baseline model, while also surpassing YOLOv9 and YOLOv10. Our method has the potential to provide a more comprehensive and detailed tool for early disease intervention in tomato cultivation.

## 2 Materials and methods

### 2.1 Materials

#### 2.1.1 Dataset

**Figure 3:**
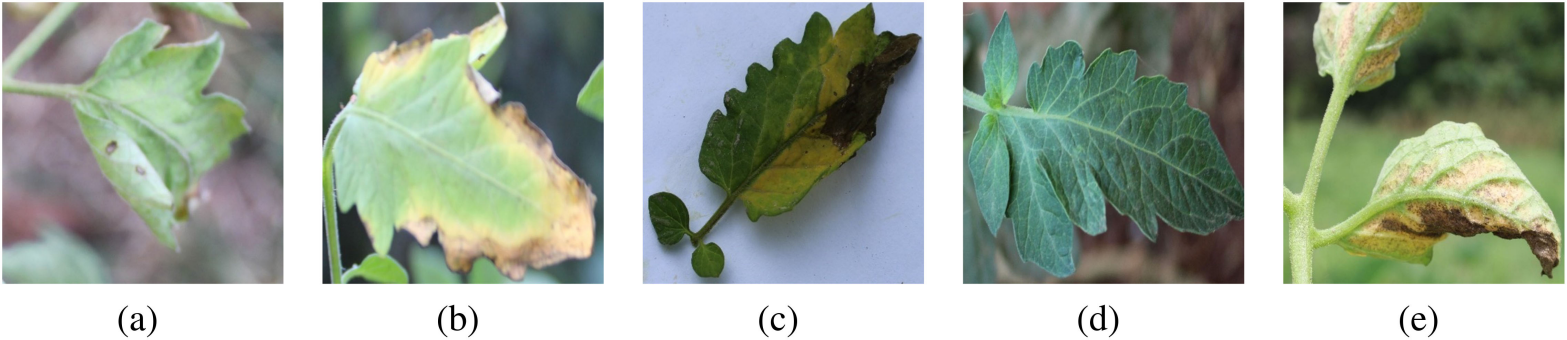
Display of five tomato disease leaves. **(a)** septoria leaf spot, **(b)** leaf blight, **(c)** leaf verticillium wilt, **(d)** healthy leaf, **(e)** leaf curl.

We evaluated our model on two public datasets: the PlantDoc plant disease detection dataset Singh et al. (2019) and the CCMT tomato disease dataset Mensah et al. (2023). The PlantDoc contains 2,598 data samples for 13 plant species and 17 disease classes. The CCMT consists of 4960 samples distributed across 5 tomato leaf disease classes. **Figure 3** presents the details of specific diseased leaves: (a) septoria leaf spot, (b) leaf blight, (c) leaf verticillium wilt, (d) healthy leaf, (e) leaf curl.

**Figure 4:**
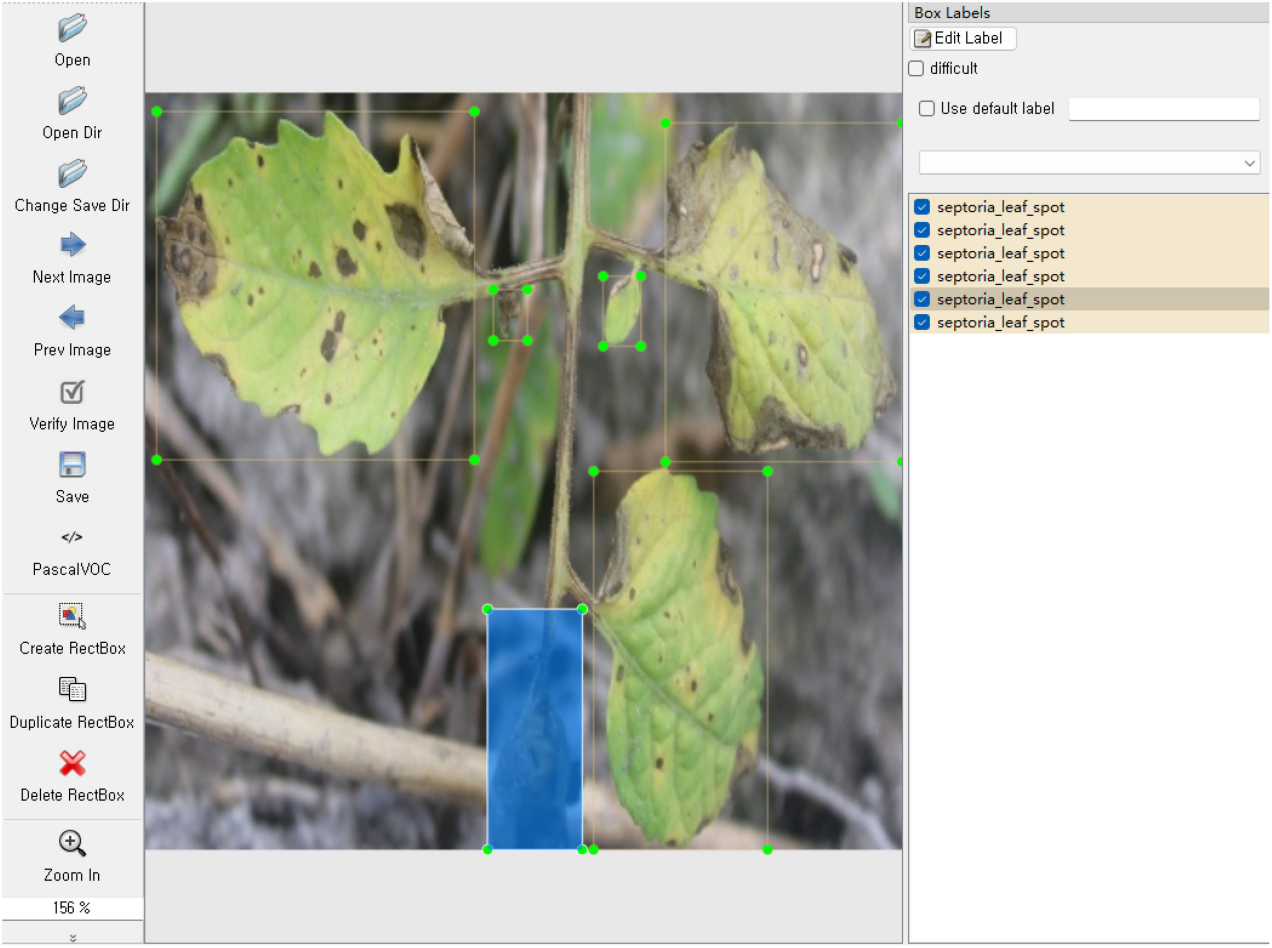
LabelImg annotation.

However, the detection targets in this tomato data sample were not annotated. Therefore, we first used the LabelImg annotation tool to annotate the 4960 images in the tomato leaf disease dataset, as shown in **Figure 4**. Then, corresponding XML files containing bounding box coordinates and category labels were generated. Subsequently, these XML files were converted into txt annotation files used by the YOLO model. After preprocessing, the dataset was partitioned into three distinct subsets: 4059 instances for training, 451 for validation, and 450 for testing. The training set served to fit the model parameters, while the validation set was employed to fine-tune hyperparameters during the learning process and conduct initial assessments of model performance. Ultimately, the test set was utilized to gauge the final model’s capacity for generalization.

#### 2.1.2 Implementation details

The models proposed in the study use PyTorch as the learning framework, and the experimental acceleration is configured with AMD and RTX 4090. During the training process, 640 × 640 images are randomly cropped from the sample images. The batch size for each GPU is set to 12. Each model undergoes a training regimen consisting of 150 iterations. The optimization process employs the Adam algorithm, with the learning rate initially set at 0.01. As shown in **Table 1**, detailed experimental setup details are presented.

**Table 1:** Our experimental environment.

Computational Ecosystem	Details
System software	64 Bit Windows 11
Coding	Python 3.9
GPU	RTX 4090
CPU	4.20 GHz AMD 16 Core Processor
Pytorch	2.2.2
CUDA	11.8

### 2.2 Methods

#### 2.2.1 Macroscopic architecture

**Figure 5:**
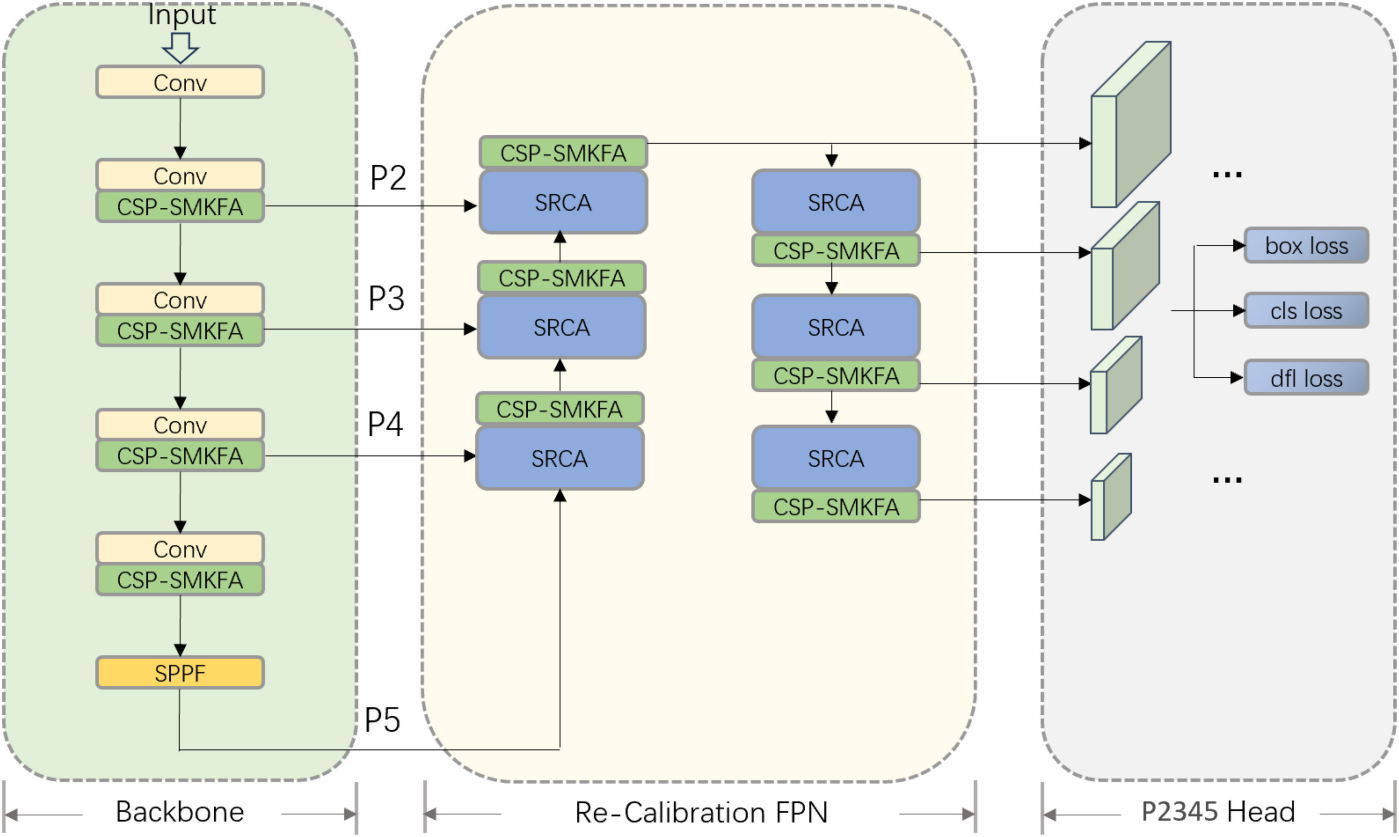
The overall architecture of TomatoLeafDet.

**Figure 5** illustrates the structural diagram of the TomatoLeafDet model architecture for detecting tomato leaf diseases. It is primarily divided into three parts: Backbone, Re-Calibration FPN, and P2345 Head. Through the introduction of new modules and optimized design, this model aims to enhance multi-scale feature extraction, fusion, and detection capabilities.

In the initial step of the Backbone section, the model replaces the original C2F module with CSPSMKFA, combined with Conv convolutional modules for feature extraction. Through multi-scale partial convolution, the CSP-SMKFA module can more effectively capture different scale information in the image, especially enhancing feature expression ability when dealing with large differences in target sizes. This improvement helps to enhance the model’s capture of input image features, providing richer semantic information for subsequent feature fusion processing. Simultaneously, the SPPF module is retained at the end of the Backbone, further processing features at multiple scales, and increasing the robustness of feature representation. Subsequently, in the Neck section, the model introduces a new Re-Calibration FPN, where the SRCA module can perform spatial recalibration on feature maps at different levels, enabling the network to more effectively select and focus on useful feature information. Combined with the CSP-SMKFA module, it enhances the effect of multi-scale feature fusion. This structure allows for more comprehensive information transmission between features at different levels, optimizing the detection performance of multi-scale targets. Finally, in the Head section, the model introduces a P2 detection head specifically for small target detection, working in conjunction with P3, P4, and P5 detection heads. This design can better capture fine-grained information in low-level feature maps, thereby improving the model’s detection accuracy for small-scale targets. In terms of loss functions, the model still employs traditional regression loss (box loss), classification loss (cls loss), and DFL loss (dfl loss) to ensure the accuracy of predicted bounding box positions and categories. Overall, the model has been optimized at the Backbone, Neck, and Head levels, particularly through the introduction of CSP-SMKFA and SRCA modules, enhancing the ability of multi-scale feature extraction and fusion. This makes the improved TomatoLeafDet model more robust and accurate in multi-scale object detection tasks.

#### 2.2.2 Cross stage partial - serial multi-kernel feature aggregation

**Figure 6:**
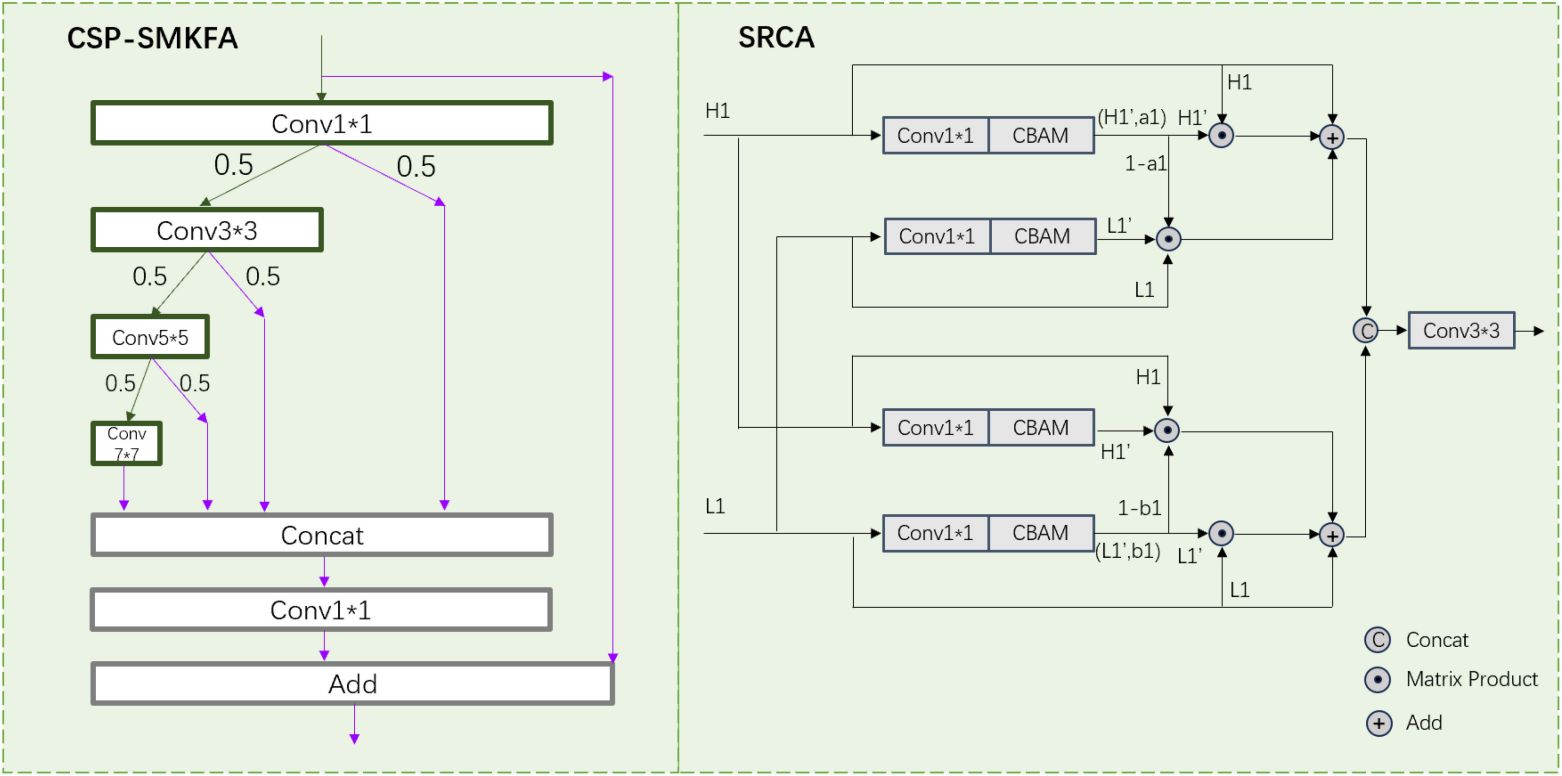
The structure of CSP-SMKFA and SRCA.


The CSP-SMKFA module adopts a novel partial multi-scale feature concatenation aggregation strategy to enhance computational efficiency and feature representation capability. As shown in **Figure 6**, its structural process is as follows: Initially, the input features undergo preliminary processing through Conv1 x 1, subsequently dividing into two branches. The main branch then employs a cascaded convolution structure: a) Conv3 x 3 extracts local features, b) Conv5 x 5 captures medium-scale contextual information, and c) Conv7 x 7 obtains global features with a larger receptive field. Each convolutional layer allocates 50% of its output channels to the next convolutional layer and skips the connection. The skip connection branch directly transmits the remaining 50% of the Conv1 x 1, Conv3 x 3, Conv5 x 5, and Conv7 x 7 outputs, preserving original feature information. A Concat operation then concatenates features of different scales (1x1, 3x3, 5x5, 7x7 convolution outputs) along the channel dimension. Subsequently, Conv1 x 1 further fuses these multi-scale features, producing a unified feature representation. Finally, a residual connection adds the original input features to the processed features, forming the final output. This design effectively integrates spatial information at different scales while reducing computational complexity through partial channel multi-scale processing. The cascaded convolution structure progressively expands the receptive field, capturing multi-scale context. Skip connections and residual connections ensure the preservation of original information, facilitating gradient propagation. The final feature aggregation and fusion steps further enhance the model’s expressive capacity, enabling it to better adapt to the demands of various multi-scale object detection tasks. Therefore, the process of CSP-SMKFA can be formulated as:

(1) $$O_{1}1^{\prime },O_{1}2^{\prime }={\text{S}}_{\left(0.5,0.5\right)}\left({\text{Conv}}_{1\times 1}\left(Input\right)\right)$$

(2) $$O_{3}1^{\prime },O_{3}2^{\prime }={\text{S}}_{\left(0.5,0.5\right)}\left({\text{Conv}}_{3\times 3}\left(O_{1}1^{\prime }\right)\right)$$

(3) $$O_{5}1^{\prime },O_{5}2^{\prime }={\text{S}}_{\left(0.5,0.5\right)}\left({\text{Conv}}_{5\times 5}\left(O_{3}1^{\prime }\right)\right)$$

(4) $$O_{7}^{\prime }=\left({\text{Conv}}_{7\times 7}\left(O_{5}1^{\prime }\right)\right)$$

(5) $$\text{Output}={\text{Conv}}_{1\times 1}\left(\text{Concat}\left(O_{1}2^{\prime },O_{3}2^{\prime },O_{5}2^{\prime },O_{7}^{\prime }\right)\right)⊕Input$$

where ${\text{S}}_{\left(0.5,0.5\right)}\left(\cdot \right)$ represents splitting the output feature map vector along the channel dimension with a ratio of 0.5 for each part. ⊕ represents addition. Concat(·) is concatenation.

#### 2.2.3 Symmetrical re-calibration aggregation

When processing targets of different scales, there is a tendency to lose significant semantic information. Shallow features contain less semantic content but have clear boundaries and rich details. Deep features, however, encompass abundant semantic information. Directly fusing shallow and deep features may result in redundant information in the fused features Han et al. (2019). To address this issue, we propose the Symmetrical Re-calibration Aggregation (SRCA) module, which utilizes a self-attention mechanism to fuse high-resolution and low-resolution features. Feature fusion is conducted selectively through weighting, capturing more comprehensive fine-grained information, and recalibrating target positions.

**Figure 6** illustrates the structure of the SRCA module, designed to effectively fuse high-resolution and low-resolution features. The module comprises four parallel complementary processing paths, each handling high-resolution (H1) and low-resolution (L1) features. For instance, in the first two paths’ processing flow: Initially, high-resolution feature H1 is processed through a 1x1 convolutional layer and Convolutional Block Attention Module (CBAM) Woo et al. (2018), yielding compressed mapped feature H1’ and attention weight a1. Low-resolution feature L1 undergoes the same processing, producing L1’. Subsequently, through a self-attention mechanism, (1-a1) is fused with L1 and L1’. Then, the fused features are added to the original H1. The latter two paths follow a similar processing flow but interchange the input positions of high and low-resolution features: First, L1 is processed through 1x1 convolution and CBAM, yielding L1’ and attention weight b1. Then, H1 undergoes the same processing to obtain H1’. Internal upsampling and downsampling functions adjust the sizes of (1-b1), H1, and H1’ for three-part fusion. The fusion result is then added to the original L1. Finally, the outputs of these four paths are channel-fused and passed through a Conv3 x 3 layer to output a rich fused feature. This design allows the model to adaptively select and fuse features from different resolutions, effectively capturing multi-scale information. By cross-processing high and low-resolution features, the module can more comprehensively utilize spatial information at different scales, thereby enhancing the richness and effectiveness of feature representation. Therefore, the process of SRCA can be formulated as:


(6) $$H_{1}^{'},a_{1}=\text{CC}\left(H_{1}\right)$$

(7) $$L_{1}^{'},b_{1}=\text{CC}\left(L_{1}\right)$$

(8) $${\text{output}}_{up}=H_{1}⊕\left(H_{1}^{'}⊙H_{1}\right)⊕\left(L_{1}^{'}⊙L_{1}⊙\left(1-a_{1}\right)\right)$$

(9) $${\text{output}}_{down}=L_{1}⊕\left(L_{1}^{'}⊙L_{1}\right)⊕\left(H_{1}^{'}⊙H_{1}⊙\left(1-a_{1}\right)\right)$$

(10) $$\text{Output}={\text{Conv}}_{3\times 3}\left(\text{Concat}\left({\text{output}}_{up},{\text{output}}_{down}\right)\right)$$

Where CC represents Conv_1×1_ and CBAM, used to process input features, halve the channels, and obtain new feature maps and weight coefficients. ⊕ represents addition. ⊙ represents matrix product. Concat(·) is concatenation.

## 3 Experiments

### 3.1 Experimental indicators

In this study, we employed multiple metrics to evaluate our model’s performance: Parameters, GFLOPs (Giga Floating Point Operations Per Second), mean Average Precision (mAP50-90), and mean Average Precision (mAP50). Among these, mAP50 was selected as the primary evaluation metric. The calculation process for mean Average Precision is delineated in Equations 11-14.


(11) $$\text{Precision}=\frac{TP}{TP+FP}$$

(12) $$\text{Recall}=\frac{TP}{TP+FN}$$

(13) $$AP=\int_{0}^{1}P\left(R\right)\;dR$$

(14) $$mAP=\frac{\sum_{i=1}^{K}AP_{i}}{K}$$

The variable K signifies the total count of distinct object classifications within the dataset, while each class’s precision is quantified by its specific Average Precision (AP) score. In the performance evaluation equations, several key indicators are utilized: True Positives (TP) represent accurately identified instances of the target condition, False Positives (FP) indicate cases where the algorithm incorrectly flagged non-existent conditions as present, and False Negatives (FN) encompass actual occurrences of the condition that the system failed to recognize.

### 3.2 Comparison studies

**Figure 7:**
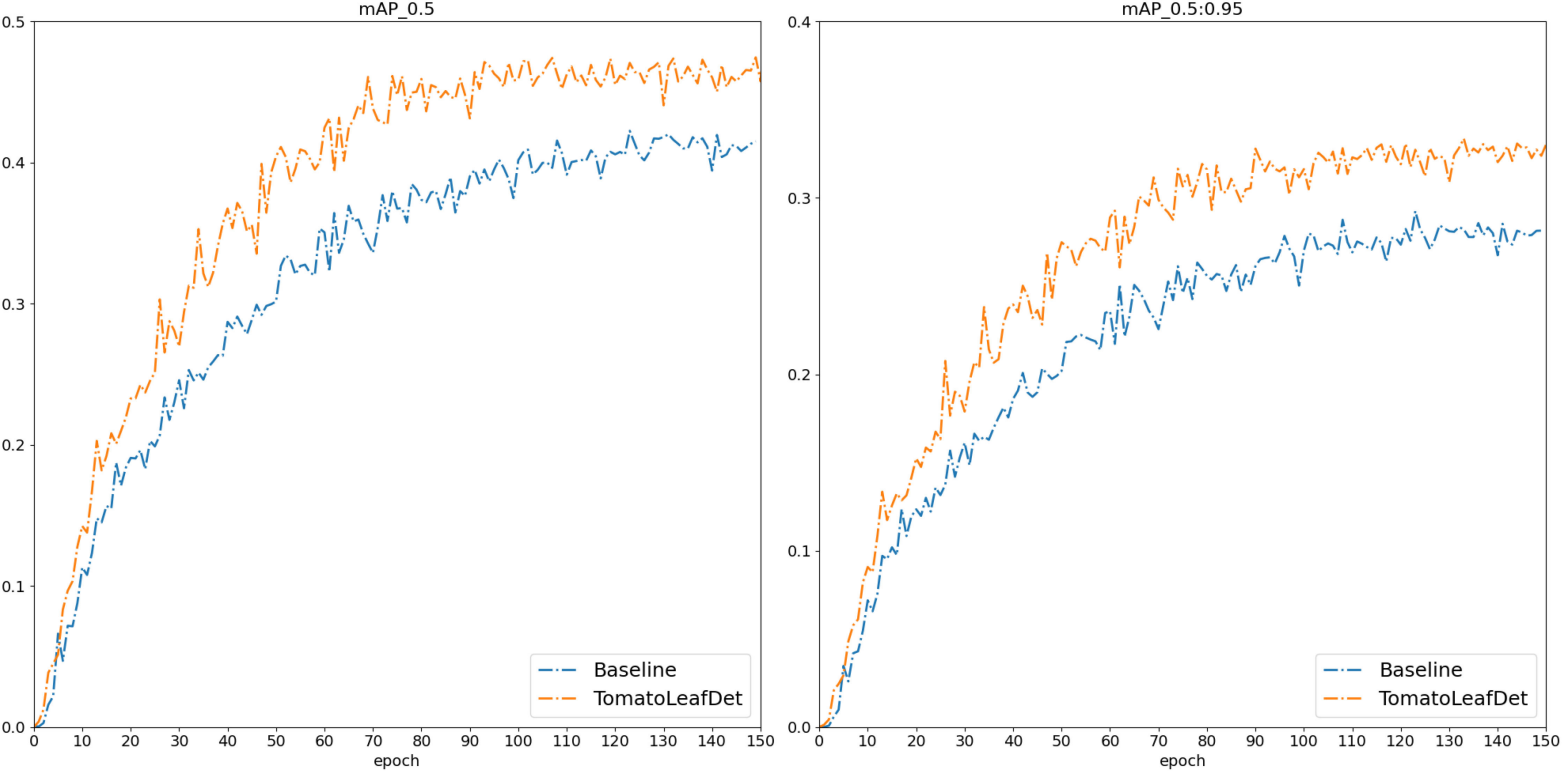
The mAP comparison between baseline and TomatoLeafDet on PlantDoc dataset.

To validate the effectiveness of the proposed model in this study, we first conducted comparative experiments on the publicly available PlantDoc dataset. We compared current mainstream object detection models, with each comparative model using the same experimental parameters. **Table 2** presents the experimental results of our proposed TomatoLeafDet model compared to other state-of-the-art real-time detection models on the PlantDoc dataset. Compared to the baseline model, TomatoLeafDet achieved improvements of 9.3% and 13.7% in mAP50 and mAP50-95, respectively. When compared to the advanced YOLOv10n, TomatoLeafDet also demonstrated a 7.2% increase in mAP50 and a 12.2% increase in mAP5095. **Figure 7** visually illustrates the performance gap between the TomatoLeafDet model and the baseline model, clearly showing that TomatoLeafDet consistently outperforms the baseline model throughout the entire training process.

**Table 2:** Comparison with advanced real-time object frameworks on the PlantDoc dataset.

Model	Parameters	GFLOPs	mAP50-95	mAP50
YOLOv8n (baseline)	3,011,498	8.1	0.282	0.420
YOLOv9t	2,628,260	10.7	0.302	0.443
YOLOv10n	2,706,116	8.3	0.286	0.428
Ours	3,351,832	9.8	0.321	0.459

**Figure 8:**
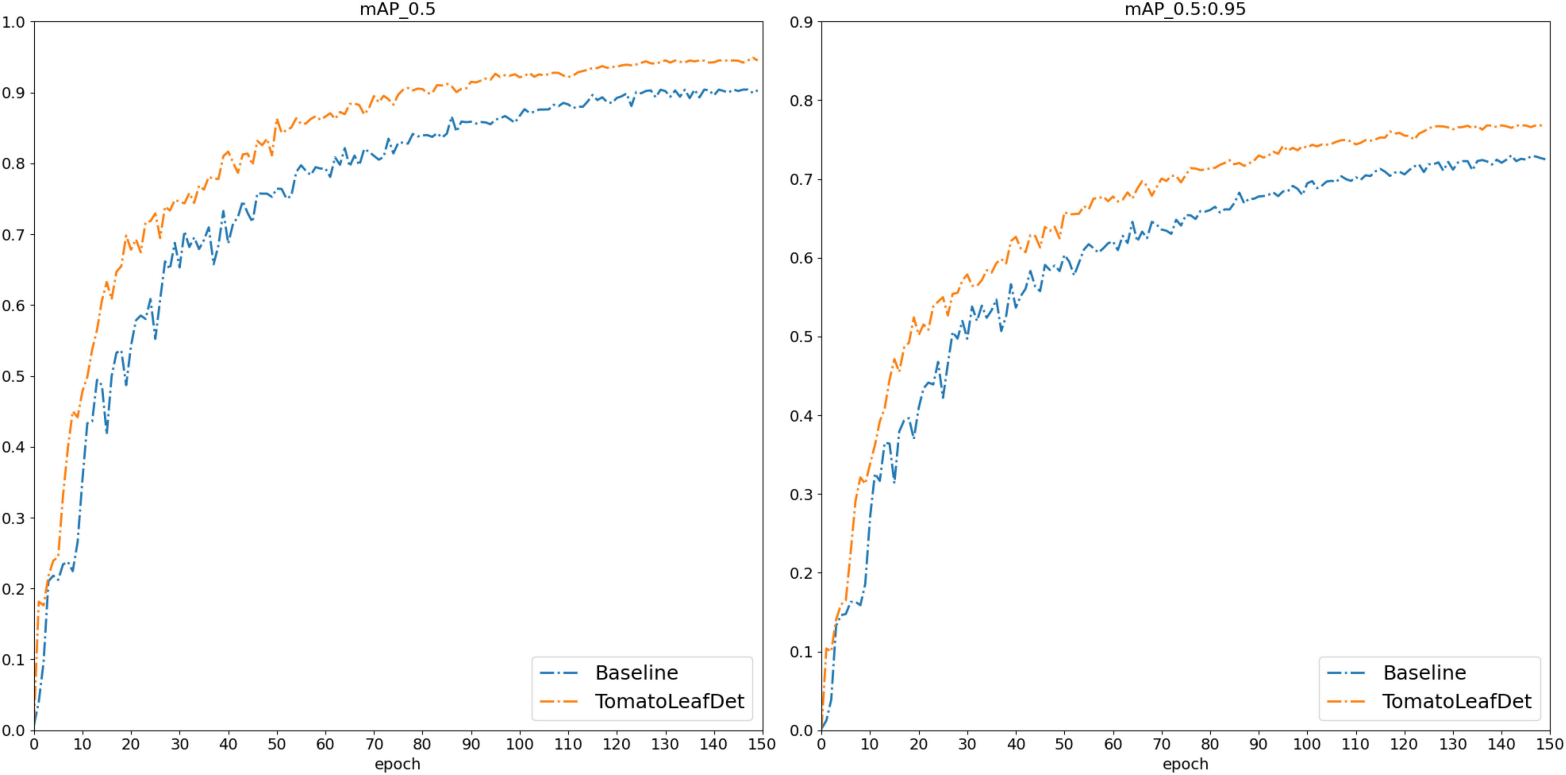
The mAP comparison between baseline and TomatoLeafDet on the CCMT tomato dataset.

After that, we conducted a comprehensive comparative evaluation of the TomatoLeafDet model on the tomato leaf disease dataset. As shown in **Table 3**, we selected the two-stage model Faster R-CNN and current mainstream single-stage YOLO series models for comparison. Notably, compared to these real-time detection models, our model demonstrated superior performance in mAP50–95 and mAP50, primarily attributed to the CSP-SMKFA and Re-CalibrationFPN multi-scale feature processing styles, which enhanced the model’s multi-scale perception capabilities. Compared to the baseline model, TomatoLeafDet achieved improvements of 4.5% and 4.4% in mAP50–95 and mAP50, respectively. Compared to the two stage Faster r-cnn model, TomatoLeafDet significantly reduced parameters and GFLOPs while improving mAP50 by 6.1%. Our model also showed significant advantages over larger single-stage models, for example, compared to YOLOv8s, TomatoLeafDet reduced parameters by approximately 70% while notably increasing mAP50 by 1.5%. Compared to YOLOv9s, TomatoLeafDet reduced GFLOPs by about 74.7% while significantly improving mAP50 by 2.0%. It is also worth noting that compared to the current advanced model YOLOv10n, our TomatoLeafDet model surpassed it by 0.021 mAP50. **Figure 8** visually illustrates the average precision advantage of our model over the baseline model throughout the entire training process.

**Table 3:** Object detection with different frameworks on CCMT tomato dataset.

Model	Parameters	GFLOPs	mAP50-95	mAP50
Faster-rcnn	137,101,141	370.0	0.670	0.888
YOLO7	3,760,872	105.5	0.641	0.853
YOLOv8n (baseline)	3,011,823	8.2	0.735	0.905
YOLOv8s	11,127,519	28.4	0.757	0.931
YOLOv9t	2,618,510	10.7	0.739	0.913
YOLOv9s	9,601,118	38.7	0.760	0.927
YOLOv10n	2,696,366	8.2	0.743	0.924
Ours	3,348,532	9.8	0.768	0.945

### 3.3 Ablation studies

TomatoLeafDet incorporates Re-CalibrationFPN, CSP-SMKFA, and P2 detection head. We conducted ablation experiments on these components sequentially, with results shown in **Table 4**. We first added the ReCalibrationFPN without the P2 head structure, which improved performance by 1.7% mAP. Subsequently, we added the P2 detection head on this basis, further increasing the mean Average Precision (mAP) by 1.1%. To verify the effectiveness of the CSP-SMKFA module, we independently added it to the baseline model, resulting in a 3.0% improvement in mAP50–90 and a 2.7% improvement in mAP50. Finally, we integrated both Re-CalibrationFPN with P2 and CSP-SMKFA into the baseline model, constructing our new model, TomatoLeafDet. As shown in the last row of **Table 4**, the combination of our proposed modules effectively improved the model’s performance by 4.5% and 4.4% in mAP50–95 and mAP50, respectively. This validates the effectiveness of our proposed Re-CalibrationFPN, CSP-SMKFA, and P2 detection head in tomato leaf disease detection.

**Table 4:** Ablation studies of key components on CCMT tomato dataset.

Model	Re-Calibration FPN without P2	Re-Calibration FPN with P2	CSP-SMKFA	Parameters	GFLOPs	mAP50-95	mAP50
1	×	×	×	3,011,823	8.2	0.735	0.905
2	✓	×	×	3,806,303	9.9	0.743	0.920
3	×	✓	×	3,761,476	10.9	0.746	0.930
4	×	×	✓	2,802,695	8.1	0.757	0.929
5	✓	×	✓	3,402,375	8.9	0.760	0.936
Ours	×	✓	✓	3,348,532	9.8	0.768	0.945

The symbol “√” indicates that the module is included, while the symbol “×” indicates that the module is removed.

### 3.4 Visual comparative studies

**Figure 9:**
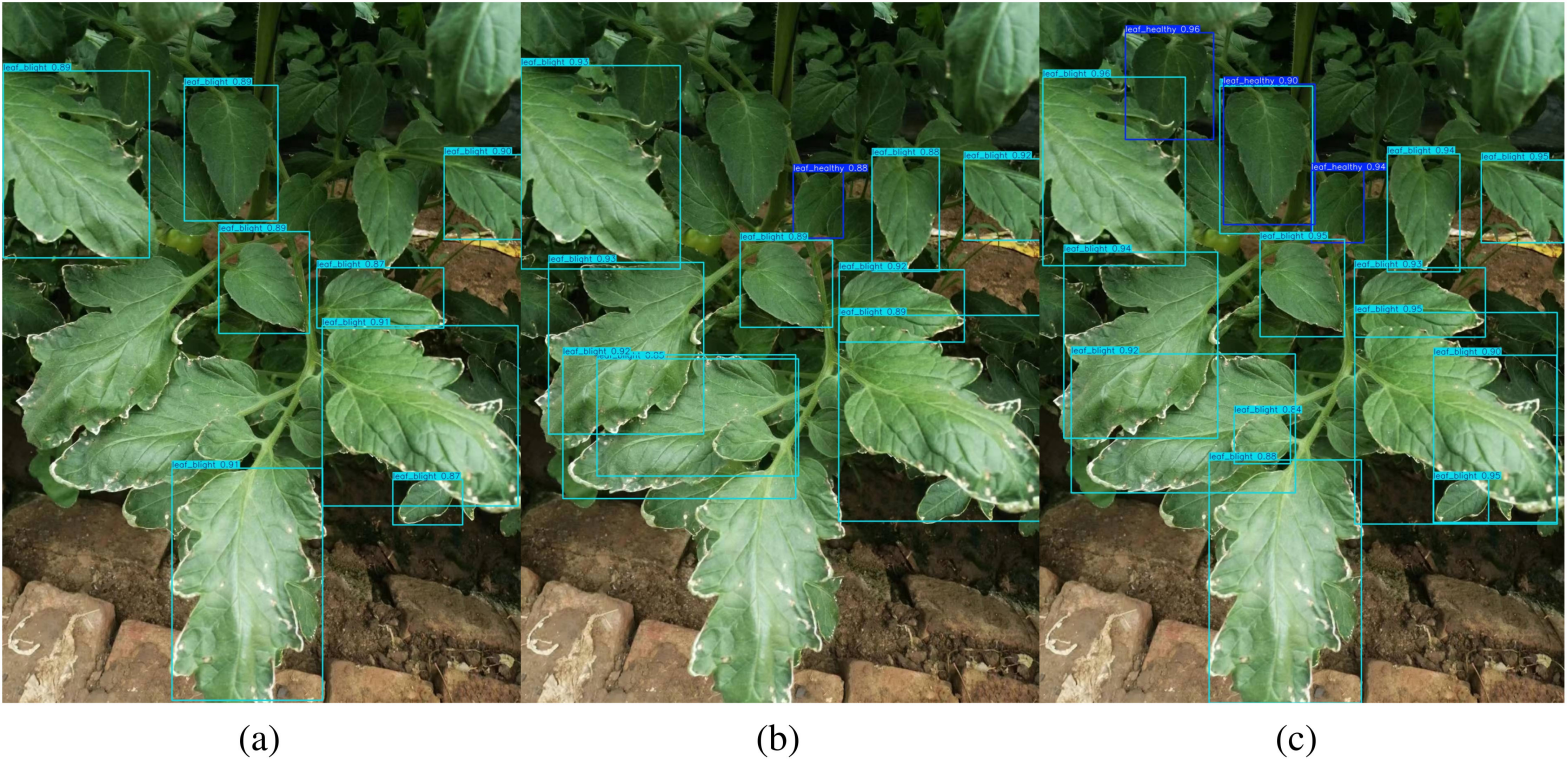
Comparison of the visualization detection result of different models, **(a)** Baseline Model; **(b)** Yolov10n Model; **(c)** TomatoLeafDet.

To visualize the advantages of TomatoLeafDet for different sizes of tomato leaves detection, the differentiated detection results of the baseline model, the advanced model yolov10n, and our proposed model on the CCMT tomato dataset are shown in **Figure 9**. It can be observed that our model exhibits increased attention and sensitivity to small-scale leaves while simultaneously detecting normal-sized leaves. In contrast, the other two models predominantly focus on normal-scale target leaves. Consequently, these results demonstrate that our proposed novel model performs excellently in tomato leaf disease detection. Therefore, it provides a potential solution for early predicting and preventing tomato leaf diseases.

## 4 Conclusion

In this study, we introduced Re-CalibrationFPN as a solution to address the limitations of the baseline model in handling multi-scale object features. It comprises two key components: CSP-SMKFA and SRCA. CSP-SMKFA utilizes concatenated multi-kernel partial convolutions to perceive multi-scale feature information. At the same time, SRCA further effectively integrates deep rich information and shallow detail information through a symmetrically complementary structure. Additionally, we incorporated a P2 small object detection head, enabling the model to focus on detailed features of small-sized objects while attending to multi-scale object features. Through extensive experimentation, we demonstrated that our proposed model achieved advanced performance on both the PlantDoc dataset and the CCMT tomato dataset, surpassing current mainstream object detection models.

Although our model has significantly advanced tomato leaf disease detection, further research is required to bridge the gap between experimental results and practical applications. In subsequent studies, we plan to collect and establish more comprehensive datasets to train and improve model performance. We will continue to address the limitations in multi-scale and small object detection while gradually considering research into lightweight models. Future research focuses on integrating lightweight detection models with drones and robots, accelerating the establishment of early prediction and prevention systems for precision vegetable cultivation.

## Data Availability

The original contributions presented in the study are included in the article/supplementary material. Further inquiries can be directed to the corresponding authors.
