# Supplementary figures and images for: A multi-scale detection model for tomato leaf diseases with small target detection head
Source: Front Plant Sci. 2025 Sep 16;16:1598534. doi: 10.3389/fpls.2025.1598534 (PMC12479537; doi:10.3389/fpls.2025.1598534)

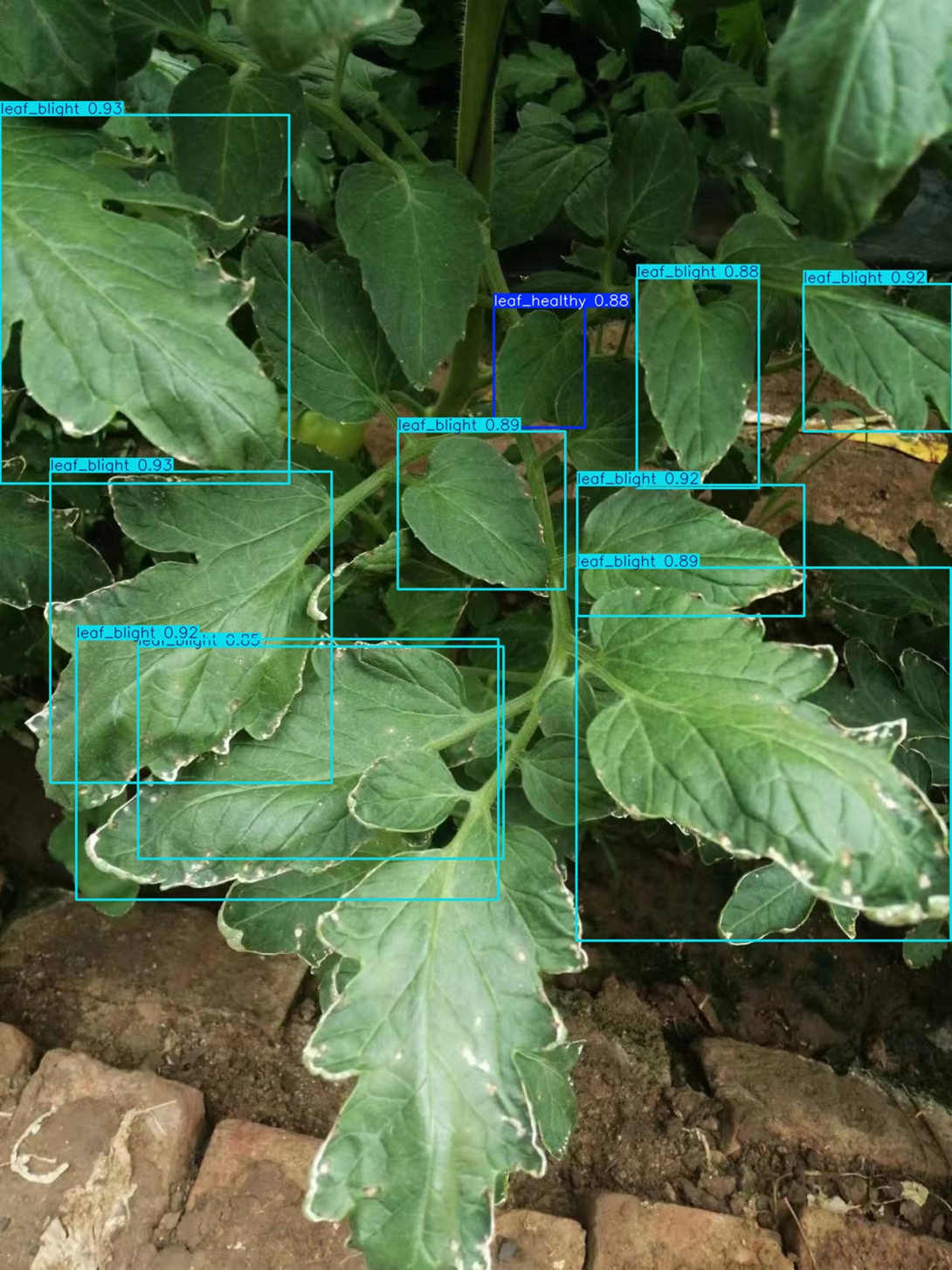

Supplement: Supplementary file 1 [file Image1.jpeg]

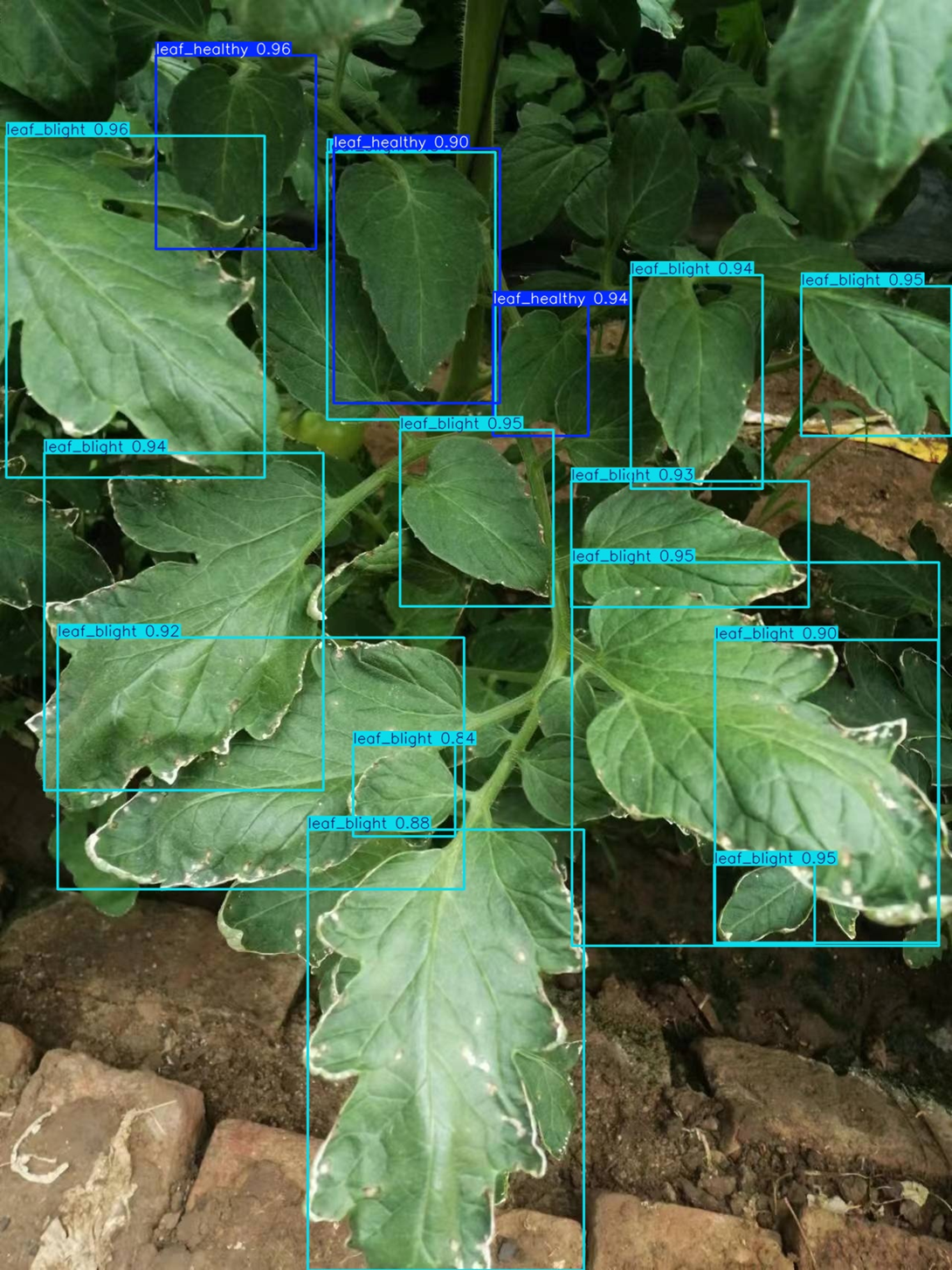

Supplement: Supplementary file 2 [file Image2.jpeg]
